# Ovulation patterns affect the offspring sex ratios and change with the women’s age

**DOI:** 10.1186/s12978-022-01462-2

**Published:** 2022-07-08

**Authors:** Misao Fukuda, Kiyomi Fukuda, Shawn Mason, Kenichi Tatsumi, Takashi Shimizu, Taiichiro Akahori, Tsunekazu Matsumoto, Masahiro Tahara, Claus Yding Andersen

**Affiliations:** 1 M&K Health Institute, 30-9 Kariya, , Ako, Hyogo 678-0239 Japan; 2 Umegaoka Women’s Hospital, 1-33-3 Umegaoka, Setagaya-ku, Tokyo, 154-0022 Japan; 3 Shimizu Women’s Clinic, 2-2-4 Minamiguchi, Takarazuka, Hyogo 665-0011 Japan; 4 Akahori Hospital, 33 Tsubakikoge, Tsuyama, Okayama 708-0051 Japan; 5 Kobe-Motomachi Yume Clinic, 44 Akashi-cho, Chuo-ku, Kobe, 650-0037 Japan; 6 Taniguchi Hospital, 1-5-20 Oonishi, Izumisano City, 598-0043 Japan; 7 Laboratory of Reproductive Biology, Juliane Marie Center, Rigshospitalet, Section 5712, University Hospital of Copenhagen, Blegdamsvej 9, 2100 Copenhagen, Denmark; 8 Present Address: Nomura Clinic, 3-2-28 Chuo-ku Takatsu, Osaka, 542-0072 Japan

**Keywords:** Women’s age, Offspring sex ratio, Right-sided ovulation, Contralateral ovulation, Left–left–right ovulation (LLR)

## Abstract

**Background:**

The aim of this study was to evaluate whether women’s ages at conception and the ratio of male to female infants are associated with various ovulation patterns.

**Methods:**

An observational clinical study was conducted in private OB/GYN clinics. Infertile women with regular menstrual cycles receiving intrauterine insemination (IUI) and/or in-vitro fertilization (IVF) had their ovulation patterns monitored in three consecutive spontaneous cycles receiving infertility treatment in the third cycle. Ovulation patterns were also observed in women with slight ovulation disorders during IUI and/or IVF in clomiphene citrate stimulated cycles. All the pregnant women’s ages at conception and their respective offspring sex ratios were compared to various ovulation patterns. Statistical evaluation was performed using ANOVA, unpaired t test, χ^2^ test or Fisher’s exact test, heterogeneity χ^2^ test, odds ratios at 95% confidence intervals and logistic regression.

**Results:**

Contralateral ovulation (i.e. ovulation jumping from ovary to the other) was more often observed in relatively younger women, who showed a higher probability of having a boy than after ipsilateral ovulation. There was a significantly higher frequency of boys being conceived following three consecutive ovulations with a left–left–right (LLR) ovulation pattern, while three ovulations from the left ovary (LLL) were associated with a higher frequency of girls. We also found two consecutive menstrual cycles the left–right (LR) ovulation pattern showed a similar significant difference compared to the left-left (LL) ovulation. Both the infertile and infertile + fertile women groups showing right-sided ovulation, regardless of age, showed significantly higher offspring sex ratio compared to left-sided ovulation, which was not observed in the group of fertile women alone.

**Conclusions:**

LLR, LR and contralateral ovulation happens more often in younger women and favors male offspring in infertile women. Right-sided ovulation favors male offspring in infertile and infertile + fertile women, which was not observed in the group of fertile women.

## Plain Language Summary

It is of interest to evaluate whether ovulation patterns change with women’s ages at conception and affect offspring sex ratios. An observational clinical study was conducted in private OB/GYN clinics. Infertile women with regular menstrual cycles receiving intrauterine insemination (IUI) and/or in-vitro fertilization (IVF) had their ovulation patterns monitored in three consecutive spontaneous cycles receiving infertility treatment in the third cycle. Ovulation patterns were also observed in women with slight ovulation disorder during IUI and/or IVF in clomiphene citrate stimulated cycles. Women’s ages and offspring sex ratios were compared to various ovulation patterns. The contralateral ovulation was more often observed in relatively younger women, who showed a higher probability of having a boy than following a pregnancy after ipsilateral ovulation. There was a significantly higher frequency of boys being conceived following three consecutive ovulations with after a left-left-right (LLR) ovulation pattern, while three ovulations from the left ovary (LLL) were associated with a higher frequency of girls. Only considering two consecutive menstrual cycles the left-right (LR) ovulation pattern showed a similar significant difference compared to the left-left (LL) ovulation. Both infertile and infertile + fertile women showing right-sided ovulation, regardless of age, showed significantly higher offspring sex ratio compared to left-sided ovulation, which was not observed in the group of fertile women alone. LLR, LR and contralateral ovulation happens more often in younger women and favor male offspring in infertile women. Right-sided ovulation favors male offspring in infertile and infertile + fertile women, which was not observed in the group of fertile women.

## Introduction

A number of stress factors have been shown to affect the male to female ratio of newborn infants. Exposure to environmental toxic agents such as dioxin [1] or tobacco smoke [2] as well as extreme natural conditions such as earthquakes [3–5] or climate change [6–8] have been shown to reduce the incidence of male infants being born. Despite the fact that the spermatozoa determine the sex of the offspring, women appear to exert a modest differential effect on the viability of male and female conceptus during development in the reproductive organs. For instance, a woman entering menarche at a relatively early age may be more likely to foster female offspring [9]. The underlying mechanism(s) that affect(s) the sex ratio is not clear.

In women ovulation will either jump from one ovary to the other (i.e. contralateral ovulation) or stay on the same side (i.e. ipsilateral ovulation) during consecutive menstrual cycles. It has previously been shown that conditions in one cycle affect the conditions and the likelihood of conception in the subsequent menstrual cycle. Ovulations jumping from one ovary to the other in two consecutive menstrual cycles (i.e. contralateral ovulation) shorten the follicular phase length [10–12] and increase the chances of conception in the latter of the two cycles as compared to ovulations from the same ovary (i.e. ipsilateral ovulation) [13–17]. Furthermore, it seems that oocytes released from the right ovary possess a higher pregnancy potential than oocytes released from the left ovary [18].

Human follicular development, from the late preantral stage to the preovulatory follicle ready to undergo ovulation, is estimated to take three menstrual cycles [19]. We have shown that an ovary being quiescent for two cycles results in an enhanced pregnancy potential of an oocyte ovulating from that ovary during the subsequent third cycle [15]. Moreover, it has been shown that in-vitro fertilization (IVF) and intrauterine insemination (IUI) treatment in which the ovulatory pattern showed two left-sided ovulations followed by one right-sided ovulation (i.e. LLR ovulation pattern) during three consecutive menstrual cycles—was associated with the highest pregnancy potential and with an increased likelihood of having a boy [20].

The present study was undertaken to evaluate whether the ovulation pattern during three consecutive menstrual cycles in women who conceive is associated with the mean age and the possibility of giving birth to a boy or girl in groups of fertile and infertile Japanese women.

## Materials and methods

Follicular development and the ovulation patterns were assessed by transvaginal ultrasound in three consecutive menstrual cycles of infertile women undergoing infertility treatment with IUI or IVF between June 1990 and July 2012. Only women who showed mono ovulation cycles were included. Those women who showed multiple ovulations were excluded Women who showed regular menstrual cycles (29.4 ± 3.1 days) without ovulation disorder underwent 420 spontaneous cycle (SP) IUI and 18 SP IVF and became pregnant. Women who showed slight ovulation disorder underwent 174 clomiphene citrate (CC) stimulated IUI cycles and 45 CC IVF cycles and became pregnant. All women irrespective of whether they received CC or not showed mono ovulation. Women receiving CC received mild ovarian stimulation consisting of 50–150 mg/day of CC (Clomid, Shionogi, Tokyo, Japan) for 5 days. All women had two intact ovaries without any ovarian cysts and none of the women received any exogenous gonadotropins for ovarian stimulation. A total of 657 singleton newborn infants were born from these 657 infertile women [age at conception: 32.8 ± 4.1 years, mean ± SD (standard deviation), range 22–42] after infertility treatments. 358 women have been added to the cohort previously reported [20]: M&K Health Institute, 19 SP IUI + 11 CC IUI + 4 CC IVF; Umegaoka Hospital, 194 SP IUI + 22 CC IUI; Akahori Hospital, 13 SP IUI + 5 CC IUI; Kobe Motomachi Yume Clinic, 1 SP IUI + 4 SP IVF; Taniguchi Hospital, 44 SP IUI + 41 CC IUI. All the attending clinics and hospitals followed the protocol as described above. The diagnoses of those 657 infertile couples were as follows: male factor, 490 couples; unknown, 167 couples. Mean ages at conception and sexes of all newborn infants were assessed according to the possible eight ovulation patterns: left–left–right (LLR), right–left–right (RLR), left–right–right (LRR), right–right–right (RRR) of right-sided ovulation, right–right–left (RRL), left–right–left (LRL), right–left–left (RLL), left–left–left (LLL) of left-sided ovulation. Considering only the previous one menstrual cycle four possible ovulation patterns occur: left–right (LR = LLR + RLR), right–right (RR = LRR + RRR), right–left (RL = RRL + LRL) and left–left (LL = RLL + LLL). We compared ages at conception and sex ratios between contralateral ovulation (LR + RL = LLR + RLR + RRL + LRL) and ipsilateral ovulation (RR + LL = LRR + RRR + RLL + LLL) and also between right-sided ovulation (LR + RR = LLR + RLR + LRR + RRR) and left-sided ovulation (RL + LL = RRL + LRL + RLL + LLL).

We compared ages at conception and offspring sex ratios between right-sided ovulation and left-sided ovulation in 684 fertile women (age at conception: 32.6 ± 4.2 years, range 21–43) who became pregnant spontaneously. These women visited M&K Health Institute or Shimizu Women’s Clinic at ~ 5–9 weeks of gestation, where the side of corpus luteum was determined by transvaginal ultrasound. The sexes of the 684 newborn infants from these fertile women were determined at birth.

The procedure of the present study was described in detail in the previous study [20]. Some parts of the present data have been used in previous reports [12–18, 20, 21]. In addition to our previous study of three consecutive cycles [20] data of women’s ages have been added and also offspring sex ratios in three consecutive cycles (i.e. LLR), two consecutive cycles (i.e. LR, contralateral ovulation) and one cycle (i.e. right-sided ovulation) of infertile women. We also included data of one cycle of fertile women.

Statistical evaluation of multiple groups of mean ages was performed using ANOVA first and when a statistical difference was detected one by one comparison was performed among multiple groups. Two groups were compared using an unpaired t test. Values were expressed as mean ± SD. Proportions were analyzed with χ^2^ test or Fisher’s exact test. When offspring sex ratios of eight ovulation patterns or of four ovulation patterns were analyzed, a heterogeneity χ^2^ test was performed first. Only if significance was detected, one by one comparison was performed. Odds ratio (ORs) of the various patterns in Tables 1 and 2 was calculated by setting LLR (in the full group of 8 patterns) or LR (in the condensed 4 ovulation pattern group) as the “control” (1.00) and comparing the number of male/female births to the other patterns in their respective groups at 95% confidence intervals (Cis). Statistical analysis of the offspring sex ratios was performed using odds ratios (ORs) and 95% confidence intervals (CIs). Differences were considered significant at *P* < 0.05.

**Table 1:** Eight possible ovulation patterns in three consecutive menstrual cycles of infertile women, mean ages at conception and associated offspring sex ratios

Ovulation pattern	Mean age^#^	No	Male/female*	Sex ratio	OR 95% CI	P-value
LLR	32.4 ± 4.2	97	69/28	2.464	1.00 control	
RLR	31.7 ± 4.1	102	53/49	1.082	0.44 0.24–0.79	0.0059
LRR	33.5 ± 4.4	87	46/41	1.122	0.46 0.25–0.84	0.0145
RRR	33.2 ± 4.0	94	52/42	1.238	0.50 0.28–0.91	0.0253
RRL	32.3 ± 3.9	96	51/45	1.133	0.46 0.25–0.83	0.0117
LRL	33.6 ± 3.6	78	42/36	1.167	0.47 0.25–0.88	0.0267
RLL	31.9 ± 3.5	64	24/40	0.600	0.24 0.12–0.48	< 0.0001
LLL	34.9 ± 4.7^$^	39	16/23	0.696	0.28 0.13–0.61	0.0016
Total	32.8 ± 4.1	657	353/304	1.161		

R: right-sided ovulation; L: left-sided ovulation

^#^ANOVA, P = 0.0002. ^$^The mean age of LLL was significantly older than that of LLR, RLR, RRR, RRL or RLL (all P < 0.05). OR 95% CI: odds ratio 95% confidence intervals. *Heterogeneity χ^2^ test: P = 0.0032

**Table 2:** Four possible ovulation patterns in two consecutive menstrual cycles of infertile women, mean ages at conception and associated offspring sex ratios

Ovulation pattern	Mean age^#^	No	Male/female*	Sex ratio	OR 95% CI	P-value
LR (LLR + RLR)	32.0 ± 4.1^$^	199	122/77	1.584	1.00 control	
RR (LRR + RRR)	33.3 ± 4.2	181	98/83	1.181	0.75 0.50–1.12	0.1766
RL (RRL + LRL)	32.9 ± 3.8	174	93/81	1.148	0.72 0.48–1.09	0.1417
LL (RLL + LLL)	33.1 ± 4.2	103	40/63	0.635	0.40 0.25–0.63	0.0003
Total	32.8 ± 4.1	657	353/304	1.161		

R: right-sided ovulation; L: left-sided ovulation

^#^ANOVA, P = 0.0254. ^$^The mean age of LR was significantly younger than that of RR, or LL (all P < 0.05). OR 95% CI: odds ratio 95% confidence intervals. *Heterogeneity × 2 test: P = 0.0032

The study protocol was approved by the institutional review board of each clinic, and all patients gave their informed consent.

## Results

The mean ages at conception and sex ratios of 657 live-born infants following IUI or IVF treatment of SP or CC according to eight possible ovulation patterns are shown in Table 1. Statistical evaluation of the 8 groups of mean ages was performed using ANOVA indicating a significant difference (P-value = 0.0002) and thus one by one comparison was performed among the 8 groups. Evaluation of the 8 groups of sex ratios was also performed using the Heterogeneity χ^2^ test which resulted in a P-value of 0.0032 and thus one by one comparison was also performed among those 8 groups as well.

The age at conception of the LLL ovulation pattern was significantly older than that of LLR, RLR, RRR, RRL or RLL (all p < 0.05) and showed a low offspring sex ratio. The LLR ovulation pattern showed the highest offspring sex ratio of 2.464 (69/28), being significantly higher than all other ovulation patterns (all p < 0.05).

An ANOVA evaluation of the condensed 4 groups of mean ages found a significant P-value of 0.0254 and thus one by one comparisons were also performed among the compacted 4 groups. Also, statistical evaluation of 4 groups of sex ratios was performed using Heterogeneity χ^2^ test producing a P-value of 0.0032 and thus one by one comparison was performed among the 4 groups. The left–right (LR) ovulation showed significantly younger age and significantly higher offspring sex ratio than left–left (LL) ovulation (Table 2).

Contralateral ovulation (LR + RL) also showed significantly younger age and significantly higher offspring sex ratio than ipsilateral ovulation (RR + LL) (Table 3).

**Table 3:** Contralateral ovulation (LR + RL) and ipsilateral ovulation (RR + LL) in two consecutive menstrual cycles of infertile women, mean ages at conception and associated offspring sex ratios

Ovulation pattern	Mean age	No	Male/female	Sex ratio	OR 95% CI	P-value
C(LR + RL)	32.4 ± 4.0^$^	373	215/158	1.361	1.00 control	
I(RR + LL)	33.2 ± 4.2^$^	284	138/146	0.945	0.69 0.51–0.95	0.0222
Total	32.8 ± 4.1	657	353/304	1.161		

C: contralateral ovulation; I: ipsilateral ovulation

^$^P = 0.0127. OR 95% CI: odds ratio 95% confidence intervals

The age at conception of right-sided ovulation was not different from that of left-sided ovulation in either infertile or fertile women. Though right-sided ovulation in fertile women did not show significantly higher offspring sex ratio than left-sided ovulation, it showed a significantly higher offspring sex ratio than left-sided ovulation in both infertile women and infertile + fertile women (Table 4).

**Table 4:** Right-sided ovulation (R) and left-sided ovulation (L) in infertile and fertile women, mean ages at conception and associated offspring sex ratios

Ovulation pattern	Mean age	No	Male/female	Sex ratio	OR 95% CI	P-value
Infertile women						
R(LR + RR)	32.6 ± 4.2	380	220/160	1.375	1.00 control	
L(RL + LL)	33.0 ± 4.0	277	133/144	0.924	0.67 0.49–0.92	0.0140
Total	32.8 ± 4.1	657	353/304	1.161		
Fertile women						
R	32.4 ± 4.3	420	217/203	1.069	1.00 control	
L	32.8 ± 4.1	264	133/131	1.015	0.95 0.70–1.29	0.7539
Total	32.6 ± 4.2	684	350/334	1.048		
Infertile + Fertile women						
R	32.5 ± 4.3	800	437/363	1.204	1.00 control	
L	32.9 ± 4.0	541	266/275	0.967	0.80 0.65–1.00	0.0498
Total	32.7 ± 4.2	1341	703/638	1.102		

Furthermore, we used logistic regression on both the full groups of 8 and the condensed groups of 4 to investigate the combined effect of age and ovulation pattern on the odds of a male being born. The results with respect to age in the group of 8 were an OR of 1.0090, along with a high P-value of 0.6445 and an OR of 1.0078, with a high P-value of 0.6888 in the case of the smaller group of 4. The results for the ovulation patterns were an OR of 0.8736, a P-value of 0.0003, a 95% CI of 0.8116–0.9404 for the group of 8 and an OR of 0.7728, P-value of 0.0006, and 95% CI 0.6668–0.8957 for the smaller group of 4.

## Discussion

This study demonstrates that the age of women who conceived in the present cohort is associated with different ovulation patterns. Three consecutive menstrual cycles on the left ovary resulted in a significantly higher age than the other ovulatory patterns, which is new information that has not previously been reported [20]. This pattern also resulted in an increased frequency of girls born when compared to other ovulatory patterns. This data confirms and extends an earlier study [20] and emphasizes that a menstrual cycle cannot be viewed as one independent entity as one menstrual cycle is affected by the activity in that particular ovary during the two previous cycles.

We found that women who conceived following a LLR ovulation pattern were significantly younger than the age of women who conceived following a LLL ovulation pattern. Furthermore, the LLR ovulation pattern favoured a higher frequency of infant boys showing a remarkably high sex ratio of 2.464, which was significantly higher than all the other ovulation patterns. This might be associated with the highest pregnancy potential of the LLR pattern [20]. In contrast, the LLL ovulation pattern was associated with the oldest average age and the possibility of having a girl.

Furthermore, when only considering two consecutive menstrual cycles, the LR ovulation pattern and contralateral ovulation resulted in a younger mean age and an augmented male offspring ratio in the group of infertile women. Moreover, right-sided ovulation favoured male offspring in the infertile and infertile + fertile women groups irrespective of age, which was, however, not observed in fertile women.

Taken together, the ovulatory pattern through three consecutive cycles appears to affect the health of the preovulatory follicles and the enclosed oocyte in ways not previously recognised and suggest that new mechanisms exert subtle effects on pregnancy potential.

This study also demonstrates that the right and left ovaries are not equally capable of producing viable oocytes with pregnancy potential. It appears that oocytes deriving in the right ovary more often result in a baby. Although the two ovaries are not vascularized in exactly the same way and therefore may receive slightly different hormonal stimulation, it is difficult to explain this difference.

The present study is unable to reveal why women at a younger age conceive when an ovulation pattern consisting of RLR or LR (i.e. contralateral ovulation) is occurring compared to other ovulation patterns such as LLL, RR or LL (i.e. ipsilateral ovulation). Additionally, no differences in mean age at conception between right and left-sided ovulations were observed. These findings are congruent with the previous report [16] indicating that the proportion of contralateral ovulation per total number of ovulations decreases with age although the proportion of right-sided ovulations per total ovulations remain almost constant, ~ around 55% irrespective of age. Moreover, the present finding may be interpreted as the offspring sex ratio decreasing with maternal age [22, 23], suggesting that the lower offspring sex ratio of reproductive aged women may be associated with more frequent ipsilateral ovulations than contralateral ovulation during advanced reproductive ages.

Weinberg et al. [24] reported an association between the sex ratio of offspring and the length of the follicular phase, indicating that a mean follicular phase length of 15.4 days in 69 women resulted in more male than female infants, whereas a mean follicular phase of 17.6 days in 64 cycles produced more females. However, no consistent pattern in the sex ratio at birth associated with the length of the follicular phase was found in a large cohort of 947 singleton live-born infants [25]. Helle [26] reported that women with long menstrual cycles tend to have more daughters (175 women and 367 daughters) supporting the follicular phase hypothesis [24]. In a cohort of 123 infertile women it was reported that the length of the follicular phase in 235 ovulation cycles in which ovulation occurred in the opposite ovary compared to the preceding cycle (15.2 ± 3.2 days) was significantly shorter than that of 175 ipsilateral ovulation cycles (15.8 ± 2.8). It was also found that contralateral ovulation favours pre-embryo development compared to ipsilateral ovulations [12], which was also seen in CC cycles [13]. The present study found that the sex ratio of infants after contralateral ovulation cycles was significantly increased than that of ipsilateral ovulation cycles. Therefore, the finding of increased proportion of females after a longer follicular phase may be associated with ipsilateral ovulations.

In some mammalian species, for instance Mongolian gerbils, more males are conceived with oocytes derived from the right ovary than those from the left ovary [27]. It has been shown that right-sided ovulation favours pregnancy more than left-sided ovulation [18]. James et al. [28] proposed that high concentrations of testosterone and oestrogen around the time of conception increase the probability of a son. The present study indicated that right-sided ovulation does not seem to show higher offspring sex ratios than left-sided ovulation in fertile women, whereas right-sided ovulation shows significantly higher offspring sex ratio compared to left-sided ovulation in infertile and infertile + fertile women. The reason for this difference is at present unknown.

The offspring sex ratio of 1.048 in fertile women is around the same as that of 1.043 in our previous study [2] while the sex ratio of 1.161 in infertile women seems to be higher than that of the general population. The reason for this difference is unknown. However, it was reported that the offspring sex ratio following infertility treatment such as IUI or IVF is high [29], which is consistent with the present results.

In the present study the percentage of non-smoking fertile couples was 32% (220/684), which was near to the value of 30% in our previous study [2]. In contrast, the percentage of non-smoking infertile couples in the present study was 45% (87/192), which was significantly (p = 0.0226) higher than that of fertile couples. Therefore, a higher percentage of non-smoking infertile couples may be one of the reasons why a higher offspring sex ratio is seen in this group of infertile couples compared to the fertile couples.

There may be some confounding factors affecting the ovulation patterns. BMI is one such possible factor. We have only BMI available for a fraction of women in this study and are therefore unable to evaluate it as a possible factor. In this study we used logistic regression to identify whether age can be a confounding factor for the ovulation patterns effect on sex ratio. We found that age had no statistically significant impact on the various patterns effect and is likely not a confounding factor.

The present study is a retrospective analysis from 1990 to 2012 covering a relatively long period. Therefore, the results may be inhomogeneous and reveal a clustering of outcomes that can be explained simply by random chance. However, we merely observed naturally occurring events of ovulation side and thus we believe these findings are unbiased.

The mechanism behind these observations is not yet clear but it may relate to levels of oestradiol and/or testosterone [30, 31] or oestradiol/androgen ratio [12, 32] at the time of conception. Also, cortisol prior to conception [33], glucose level at implantation [34] and/or adrenal androgen during pregnancy [35] may be associated with offspring sex ratios. Further research is needed to discover the reasons for these differences.

## Conclusion

LLR, LR and contralateral ovulation patterns in younger women are likely to favour male offspring. Right-sided ovulation regardless of age was found to favour male offspring in the group of infertile women as well as in the mixed group of infertile and fertile women but this tendency was not observed in the group comprised of solely fertile women. In contrast, LLL, LL and ipsilateral ovulation patterns in older women are more likely to result in female offspring. Taken together, this study's results suggest that events taking place in the ovary during previous cycles have an impact on the health of oocyte from the preovulatory follicle and may even affect the likelihood of giving birth to either a boy or a girl.

## Data Availability

Not applicable.

## Abbreviations

OB/GYN: Obstetrical and Gynecological
IUI: Intrauterine insemination
IVF: In-vitro fertilization
SP: Spontaneous cycle
CC: Clomiphene citrate
LLR: Left–left–right ovulation
RLR: Right–left–right ovulation
LRR: Left–right–right ovulation
RRR: Right–right–right ovulation
RRL: Right–right–left ovulation
LRL: Left–right–left ovulation
RLL: Right-left–left ovulation
LLL: Left–left–left ovulation
LR: Left–right ovulation
RR: Right–right ovulation
RL: Right–left ovulation
LL: Left–left ovulation
C: Contralateral ovulation
I: Ipsilateral ovulation
R: Right-sided ovulation
L: Left-sided ovulation
ANOVA: Analysis of variance
SD: Standard deviation
OR: Odds ratio
CI: Confidence interval

## References

[1] Mocarelli P, Brambilla P, Gerthoux PM, Patterson DG, Needham LL (1996). Change in sex ratio with exposure to dioxin. Lancet.

[2] Fukuda M, Fukuda K, Shimizu T, Yding Andersen C, Byskov AG (2002). Parental periconceptional smoking and male: female ratio of newborn infants. Lancet.

[3] Fukuda M, Fukuda K, Shimizu T, Møller H (1998). Decline in sex ratio at birth after Kobe earthquake. Hum Reprod.

[4] Fukuda M, Fukuda K, Mason S, Shimizu T, Yding AC (2018). The sex ratio at birth after recent major earthquakes in Japan. Early Hum Dev.

[5] Fukuda M, Fukuda K, Mason S, Shimizu T, Andersen CY (2020). Effects of earthquakes and other natural catastrophic events on the sex ratio of newborn infants. Early Hum Dev.

[6] Fukuda M, Fukuda K, Shimizu T, Nobunaga M, Mamsen LS, Yding Andersen C (2014). Climate Change is associated with male: female ratios of fetal deaths and newborn infants in Japan. Fertil Steril.

[7] Fukuda M, Shimizu T, Yding Andersen C. Climate change, Tohoku earthquake and sex ratio of fetal death & birth. The male to female ratio of newborn infants in Japan in relation to climate change, Tohoku earthquake and fetal deaths. Lap Lambert Academic Publishing 2015; 1–81

[8] Fukuda M, Fukuda K, Fukuda M (2020). The male to female ratio of newborn infants in Japan in relation to climate change, earthquakes, fetal deaths, and singleton male and female birth weights. Early Hum Dev.

[9] Fukuda M, Fukuda K, Shimizu T, Nobunaga M, Byskov AG, Andersen CY (2011). The sex ratio of offspring is associated with the mothers’ age at menarche. Hum Reprod.

[10] Wallach EE, Virutamasen P, Wright KH (1973). Menstrual cycle characteristics and side of ovulation in the Rhesus monkey. Fertil Steril.

[11] Potashnik G, Insler V, Meizner I (1987). Frequency, sequence, and side of ovulation in women menstruating normally. Br Med J.

[12] Fukuda M, Fukuda K, Yding Andersen C, Byskov AG (1996). Contralateral selection of dominant follicle favours pre-embryo development. Hum Reprod.

[13] Fukuda M, Fukuda K, Yding Andersen C, Byskov AG (1998). Contralateral ovulation shortens follicular phase length and favours pre-embryo development during ovarian stimulation with clomiphene citrate. Hum Reprod.

[14] Fukuda M, Fukuda K, Yding Andersen C, Byskov AG (1999). Anovulations in an ovary during two menstrual cycles enhance the pregnancy potential of oocytes matured in that ovary during the following third cycle. Hum Reprod.

[15] Fukuda M, Fukuda K, Yding Andersen C, Byskov AG (2000). Does anovulation induced by oral contraceptives favor pregnancy during the following two menstrual cycles?. Fertil Steril.

[16] Fukuda M, Fukuda K, Yding Andersen C, Byskov AG (2001). Characteristics of human ovulation in natural cycles correlated to age and achievement of pregnancy. Hum Reprod.

[17] Fukuda M, Fukuda K, Yding Andersen C, Byskov AG (2006). Ovulation jumping from the left to the right ovary in two successive cycles may increase the chances of pregnancy during intrauterine insemination and/or in vitro fertilization natural cycles. Fertil Steril.

[18] Fukuda M, Fukuda K, Yding Andersen C, Byskov AG (2000). Right-sided ovulation favours pregnancy more than left-sided ovulation. Hum Reprod.

[19] Gougeon A (1986). Dynamics of follicular growth in the human: a model from preliminary results. Hum Reprod.

[20] Fukuda M, Fukuda K, Tatsumi K, Shimizu T, Nobunaga M, Byskov AG, Yding Andersen C (2011). The ovulation pattern during three consecutive menstrual cycles has a significant impact on pregnancy rate and sex of the offspring. Fertil Steril.

[21] Fukuda M, Fukuda K, Byskov AG, Yding AC (2011). Do hormonal characteristics of the luteal phase affect the conception rate of women undergoing IUI treatment in the following menstrual cycle?. Reprod Biol Insights.

[22] Juntunen KS, Kvist AP, Kauppila AJ (1997). A shift from a male to a female majority in newborns with the increasing age of grand grand multiparous women. Hum Reprod.

[23] Matsuo K, Ushioda N, Udoff LC (2009). Parental aging synergistically decreases offspring sex ratio. J Obstet Gynaecol Res.

[24] Weinberg CR, Baird DD, Wilcox AJ (1995). The sex of the baby may be related to the length of the follicular phase in the conception cycle. Hum Reprod.

[25] Gray RH, Simpson JL, Bitto AC, Queenan JT, Li C (1998). Sex ratio associated with timing of insemination and length of the follicular phase in planned and unplanned pregnancies during use of natural family planning. Hum Reprod.

[26] Helle S (2009). Women with long menstrual cycles have more daughters. Epidemiology.

[27] Clark MM, Galef BG (1990). Sexual segregation in the left and right horns of the gerbil uterus: the male embryo is usually on the right, the female on the left' (Hippocrates). Dev Psychobiol.

[28] James WH (1996). Evidence that mammalian sex ratios at birth are partially controlled by parental hormone levels at the time of conception. J Theor Biol.

[29] Maalouf WE, Mincheva MN, Campbell BK, Hardy IC (2014). Effects of assisted reproductive technologies on human sex ratio at birth. Fertil Steril.

[30] James WH (2001). Side of ovulation, hormones and sex ratios. Hum Reprod.

[31] Fukuda M, Fukuda K, Yding Andersen C, Byskov AG (2001). Side of ovulation, hormones and sex ratios. Hum Reprod.

[32] Yding Andersen C (1993). Characteristics of human follicular fluid associated with successful conception after in vitro fertilization. J Clin Endocnnol Metab.

[33] Chason RJ, McLain AC, Sundaram R, Chen Z, Segars JH, Pyper C, Buck Louis GM (2012). Preconception stress and the secondary sex ratio: a prospective cohort study. Fertil Steril.

[34] Larson MA, Kimura K, Kubisch HM, Roberts RM (2001). Sexual dimorphism among bovine embryos in their ability to make the transition to expanded blastocyst and in the expression of the signaling molecule IFN-tau. Proc Natl Acad Sci USA.

[35] James WH (2015). Hypothesis: High levels of maternal adrenal androgens are a major cause of miscarriage and other forms of reproductive suboptimality. J Theor Biol.

